# Improved Irritative Voiding Symptoms 3 Years after Stereotactic Body Radiation Therapy for Prostate Cancer

**DOI:** 10.3389/fonc.2014.00290

**Published:** 2014-10-21

**Authors:** Zaker Rana, Robyn A. Cyr, Leonard N. Chen, Brian S. Kim, Rudy A. Moures, Thomas M. Yung, Siyuan Lei, Brian T. Collins, Simeng Suy, Anatoly Dritschilo, John H. Lynch, Sean P. Collins

**Affiliations:** ^1^Department of Radiation Medicine, Georgetown University Hospital, Washington, DC, USA; ^2^Department of Urology, Georgetown University Hospital, Washington, DC, USA

**Keywords:** prostate cancer, SBRT, CyberKnife, IPSS, irritative, overactive bladder

## Abstract

**Background**: Irritative voiding symptoms are common in elderly men and following prostate radiotherapy. There is limited clinical data on the impact of hypofractionated treatment on irritative voiding symptoms. This study sought to evaluate urgency, frequency, and nocturia following stereotactic body radiation therapy (SBRT) for prostate cancer.

**Methods**: Patients treated with SBRT monotherapy for localized prostate cancer from August 2007 to July 2011 at Georgetown University Hospital were included in this study. Treatment was delivered using the CyberKnife^®^ with doses of 35–36.25 Gy in five fractions. Patient-reported urinary symptoms were assessed using the International Prostate Symptom Score (IPSS) before treatment and at 1, 3, 6, 9, and 12 months post-treatment and every 6 months thereafter.

**Results**: Two hundred four patients at a median age of 69 years received SBRT with a median follow-up of 4.8 years. Prior to treatment, 50.0% of patients reported moderate to severe lower urinary tract symptoms (LUTS) and 17.7% felt that urinary frequency was a moderate to big problem. The mean prostate volume was 39 cc and 8% had prior procedures for benign prostatic hyperplasia. A mean baseline IPSS-irritative (IPSS-I) score of 4.8 significantly increased to 6.5 at 1 month (*p* < 0.0001), however returned to baseline at 3 months (*p* = 0.73). The IPSS-I score returned to baseline in 91% of patients by 6 months and 96% of patients by 2 years. Transient increases in irritative voiding symptoms were common at 1 year. The mean baseline IPSS-I score decreased to 4.4 at 24 months (*p* = 0.03) and 3.7 at 36 months (*p* < 0.0001). In men with moderate to severe LUTS (IPSS ≥ 8) at baseline, the mean IPSS-I decreased from a baseline score of 6.8–4.9 at 3 years post-SBRT. This decrease was both statistically (*p* < 0.0001) and clinically significant (minimally important difference = 1.45). Only 14.6% of patients felt that urinary frequency was a moderate to big problem at 3 years post-SBRT (*p* = 0.23).

**Conclusion**: Treatment of prostate cancer with SBRT resulted in an acute increase in irritative urinary symptoms that peaked within the first month post-treatment. Irritative voiding symptoms returned to baseline in the majority of patients by 3 months post-SBRT and were actually improved from baseline at 3 years post-SBRT.

## Background

Irritative voiding symptoms are a common problem of male aging (1). In men >75 years old, the prevalence of these symptoms may be as high as 40% (2). Comorbidities may increase the risk of irritative voiding symptoms (3). They commonly develop or worsen following prostate external beam radiation therapy (EBRT) and may adversely affect a patient’s quality of life (4, 5). Patients report the development of urinary urgency, urinary frequency, and nocturia days to weeks after the start of treatment and generally resolve weeks to months following completion of EBRT. Treatment related factors such as utilization of brachytherapy (6) may impact the risk of irritative voiding symptoms. Anti-cholinergic medications may decrease these symptoms (6), but are commonly discontinued due to associated dry mouth and constipation (7).

Urinary urgency is defined as the complaint of a sudden compelling desire to pass urine, which is difficult to defer (8). Urgency may promote urinary incontinence in patients with pelvic floor muscle weakness and/or poor mobility. Urinary frequency and nocturia may increase by reducing voiding intervals (9). Nocturia is defined as a self-report of two or more voiding episodes nightly (10, 11). It causes sleep loss, daytime fatigue, and depression, which adversely affects an individual patient’s quality of life (12). In the elderly, nocturia may even increase the incidence of falls (13, 14).

Stereotactic Body Radiation Therapy (SBRT) is a safe and effective treatment for clinically localized prostate cancer (15–19). The larger dose per fraction utilized in SBRT offers the potential radiobiological benefits of hypofractionation (20). The low PSA nadirs obtained with SBRT (21) suggest it is an ablative procedure, which eradicates both cancerous and normal epithelium. The size of the prostate decreases by 35% within the first 2 years following the completion of SBRT (22). Initial reports suggest that the incidence of acute irritative voiding symptoms following SBRT is comparable to other external radiotherapy modalities, and may be less than brachytherapy (15–17). The goal of this study is to report the incidence and prevalence of irritative voiding symptoms following SBRT for clinically localized prostate cancer.

## Materials and Methods

### Patient selection

Georgetown University Hospital established its Prostate SBRT Program in 2006. As of June 2014, 750 prostate cancer patients have been treated with SBRT. At the inception of the program, a prospective database was established to record baseline patient characteristics. At each follow-up visit, toxicity and quality of life data have also been prospectively collected and recorded. Patients eligible for this study had SBRT without supplemental conventional radiation therapy for clinically localized prostate cancer and a minimum of 3 years of follow-up. Internal Review Board (IRB) approval was obtained for retrospective review of the database.

### SBRT treatment planning and delivery

Stereotactic body radiation therapy treatment planning and delivery were conducted as previously described (23, 24). Briefly, four to six stranded gold fiducials (1013-2-2, Best Medical International, Inc., Springfield, VA, USA) were placed into the prostate with two to three needle applicators via a transrectal or transperineal approach. Fused computed tomography (CT) and magnetic resonance (MR) images were used for treatment planning. The clinical target volume (CTV) included the prostate and the proximal seminal vesicles. The planning target volume (PTV) equaled the CTV expanded 3 mm posteriorly and 5 mm in all other dimensions. The prescription dose was 35–36.25 Gy to the PTV delivered in five fractions of 7–7.25 Gy over 1–2 weeks. The prescription isodose line was limited to ≥75%, which limited the maximum prostatic urethra dose to 133% of the prescription dose. Bladder volume receiving 37 Gy was limited to <5 cc. The bladder dose-volume histogram (DVH) goals were for <40% of the bladder volume to receive 50% of the prescribed dose and <10% to receive 100% of the dose. The membranous urethra was contoured and evaluated with DVH analysis during treatment planning using Multiplan (Accuray Inc., Sunnyvale, CA, USA). The DVH goal was for <50% of the membranous urethra to receive 37 Gy. To minimize the risk of local recurrence, the dose to the prostatic urethra was not constrained (25). Prostate position was verified during treatment using paired, orthogonal x-ray images (26).

### Follow-up and statistical analysis

Prospective quality of life data was obtained on the first day of SBRT treatment and during routine follow-up visits every 3 months for the first year and every 6 months for the second and third years. Patient-reported irritative voiding symptoms were assessed via the International Prostate Symptom Score (IPSS), a validated questionnaire where higher scores indicate more severe symptoms (27). The IPSS includes three questions related to irritative voiding symptoms (frequency, urgency, and nocturia). For the frequency and urgency questions, the responses were grouped into four clinically relevant categories (never, less than half the time, half or more than half the time, and almost always). For the nocturia question, the responses were grouped into four clinically relevant categories (none, 1 time, 2 times, and ≥3 times). As previously reported, nocturia was defined as urinating two or more times per night (2). The IPSS-irritative (IPSS-I) subscore has been previously defined as the sum of the scores for questions 2, 4, and 7 (28). Overall IPSS-I scores range from 0 to 15. IPSS-I resolution was defined as a return to within one point of the baseline score (29). Bother with urinary frequency was assessed via Question 4e of the Expanded Prostate Index Composite (EPIC)-26 (30), for which responses were grouped into three clinically relevant categories (no problem, small problem, and moderate to big problem).

Wilcoxon signed-rank test and Student *t*-test were used to assess the differences in ongoing toxicity and quality of life scores in comparison to baseline. To limit the effect of attrition bias, statistical analysis was limited to time points in which ≥80% of patient data were available. Sample medians and ranges were used to describe continuous variables. Actuarial likelihood estimates for time to IPSS-I resolution were determined using the Kaplan–Meier method. To statistically compare changes between time points, the levels of responses were assigned a score and the significance of the mean changes in the scores was assessed by the Wilcoxon signed-rank test. Binary logistic regression was used in the multivariate analysis to search for possible predicting factors for IPSS-I improvement. The endpoint for this analysis was an IPSS-I score at least one point lower than baseline at 3 years post-SBRT. Baseline characteristics including age, race, Charlson Comorbidity Index (CCI), risk group, partner status, work status, prostate volume, baseline α_1A_ inhibitor use, baseline androgen deprivation therapy (ADT) use, previous history of transurethral resection of the prostate (TURP), and treatment dose were included as variables in the logistic regression model. The minimally important difference (MID) in IPSS-I score was defined as a change of one-half standard deviation (SD) from the baseline (31). A non-paired Student *t*-test was used to determine if the magnitude of changes in the IPSS-I score was significantly different between men with baseline mild lower urinary tract symptoms (LUTS) (IPSS < 8) and moderate to severe LUTS (IPSS ≥8) (32).

## Results

**Figure 1:**
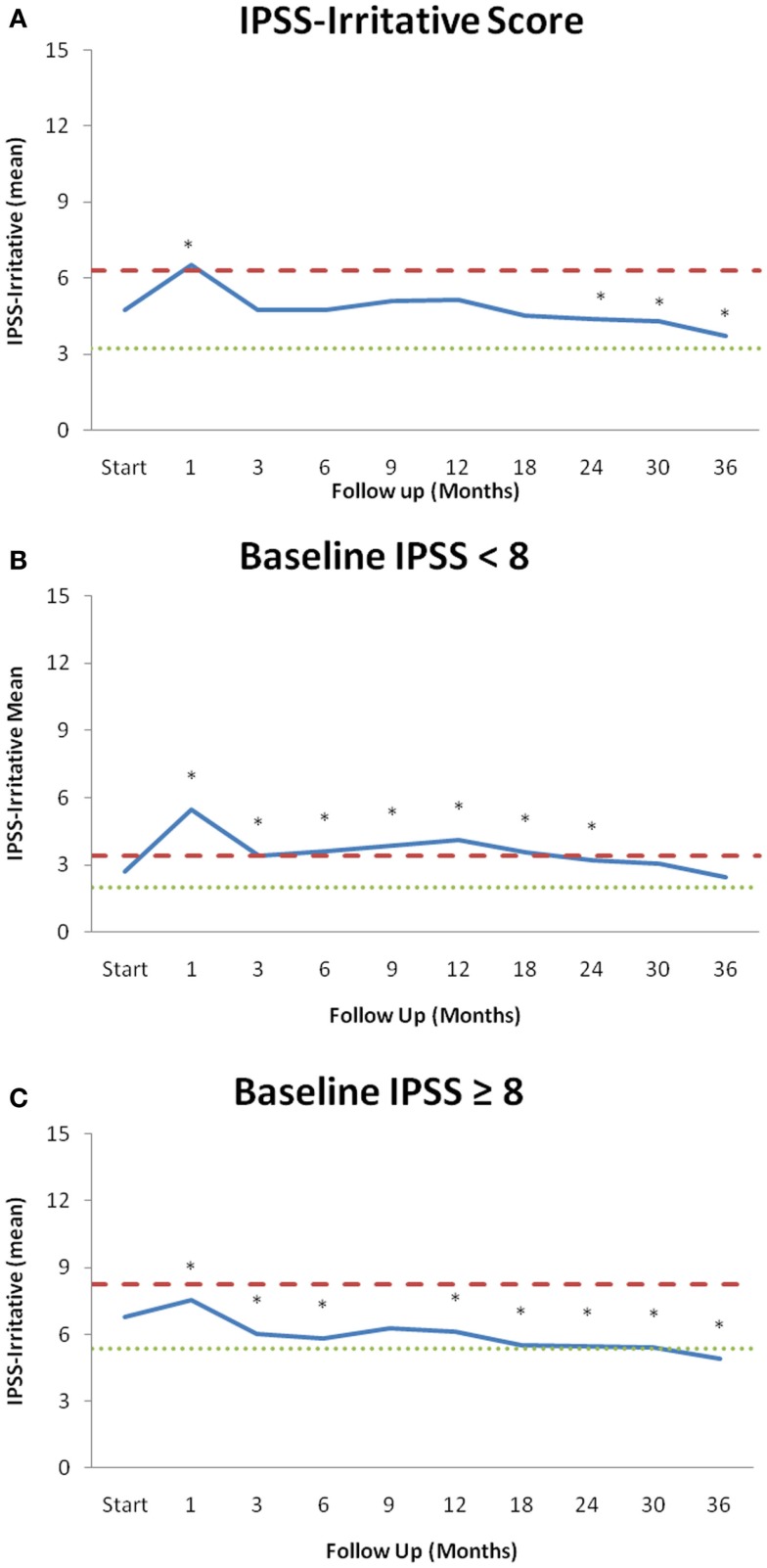
**IPSS-irritative subscores over the 36 months of follow-up stratified by baseline LUTS**. **(A)** Mean IPSS-irritative score for all patients. **(B)** Mean values of patients with a mild baseline LUTS (IPSS < 8, *n* = 102). **(C)** Mean values of patients with a moderate to severe baseline LUTS (IPSS ≥ 8, *n* = 102). Changes in scores that are statistically significant different from baseline are marked with an asterisk (*). Thresholds for clinically significant changes in scores (1/2 standard deviation above and below the baseline) are marked with dashed lines. IPSS-irritative scores range from 0 to 15 with lower values representing a more favorable outcome.

**Figure 2:**
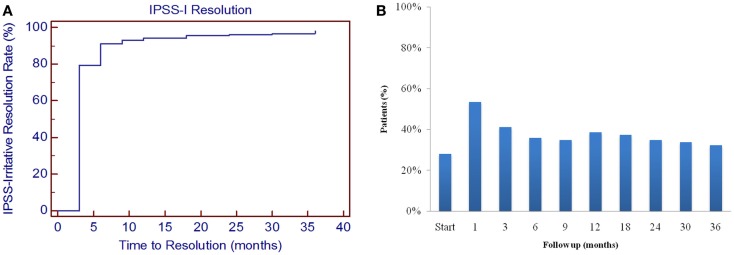
**Irritative voiding symptoms following SBRT for prostate cancer**. **(A)** Time to IPSS-I resolution was determined by the number of months it took for the IPSS-I score to return to within one point of the baseline score. **(B)** Percent of patients utilizing alpha-antagonists at each time point.

From February 2008 to July 2011, 204 prostate cancer patients were treated per our institutional SBRT monotherapy protocol with a median follow-up of 4.8 years. They were ethnically diverse with a median age of 69 years (range, 48–90 years) (Table 1). The median prostate volume was 39 cc and 8% of patients had prior procedures for benign prostatic hyperplasia (BPH). The median baseline IPSS was 8 and 28% of patients were using alpha-antagonists prior to SBRT. By D’Amico classification, 40% patients were low-, 52% intermediate-, and 8% high-risk. Fourteen percent of patients initiated ADT 3 months prior to the start of SBRT and for a mean duration of 5.5 months and a median duration of 3 months. Eighty-eight percent of patients were treated with 36.25 Gy in five 7.25 Gy fractions. The majority of patients had irritative voiding symptoms prior to treatment with a mean baseline IPSS-I score of 4.8 (Table 2; Figure 1A). At 1 month post-SBRT, the mean IPSS-I significantly increased to 6.5 (*p* < 0.0001), but returned to baseline at 3 months (*p* = 0.7319) (Table 2; Figure 1A). This increase was of borderline clinical significance (MID = 1.5). The median time to IPSS-I normalization was 3 months (Figure 2). The IPSS-I returned to baseline in 91% of patients by 6 months and 96% of patients by 2 years. At 2 years, the mean IPSS-I was close to baseline at 4.4 (*p* < 0.03). The mean IPSS-I significantly decreased from a baseline score of 4.8–3.7 (*p* < 0.0001) at 3 years post-SBRT. At 3 year post-SBRT, 59% of patients had an IPSS-I score that was less than their baseline. No baseline patient or treatment characteristics were significantly associated with improved irritative voiding symptoms on univariate or multivariate analysis 3 years following SBRT (data not shown). Alpha-antagonist utilization peaked 1 month post-treatment, at 53% patient utilization, then slowly decreased to near baseline, with 32% of patients reporting use at 3 years (Figure 2B).

**Table 1:** **Baseline patient and treatment characteristics**.

		Patients (*N* = 204) (%)
Age (y/o)	Median 69 (48~90)	
	Age ≤ 60	12.7
	60 < Age ≤ 70	46.6
	Age > 70	40.7
Race	White	54.4
	Black	38.7
	Other	7.8
Charlson comorbidity index	CCI = 0	65.2
	CCI = 1	21.1
	CCI ≥ 2	13.7
Body mass index (BMI)	Median 27.60 (15.02–44.96)	
	BMI ≥ 30	30.5
Partner status	Married or partnered	76.0
	Not partnered	24.0
Employment status	Working	48.0
	Not working	52.0
Median prostate volume (cc)	Median 39 (11.6–138.7) cc	
Procedure for BPH		7.8
α_1A_ Inhibitor usage		27.9
Risk groups (D’Amico’s)	Low	39.7
	Intermediate	52.0
	High	8.3
ADT		14.2
SBRT dose	36.25 Gy	87.7
	35 Gy	12.3
Baseline IPSS score	Median = 7.5 (0–33)	
	Mild (0–7)	50.0
	Moderate (8–19)	43.6
	Severe (≥20)	6.4

**Table 2:** **IPSS-irritative subscores**.

	Start	1	3	6	9	12	18	24	30	36
**(A)**
Mean	4.76	6.53	4.72	4.75	5.10	5.14	4.53	4.40	4.29	3.71
*p*-Value		<0.0001*	0.73	0.78	0.29	0.12	0.21	0.03*	0.003*	<0.0001*
STDEV	3.08	3.25	2.86	2.91	3.24	3.35	3.23	3.31	3.10	3.40
MID	1.54									
**(B)**
Mean	2.69	5.49	3.40	3.64	3.85	4.11	3.55	3.20	3.07	2.48
*p*-Value		<0.0001*	0.0004*	0.0013*	0.0002*	<0.0001*	0.0042*	0.02*	0.23	0.43
STDEV	1.42	2.78	2.04	2.23	2.51	3.08	2.45	2.40	2.15	2.22
MID	0.71									
**(C)**
Mean	6.80	7.56	6.01	5.81	6.29	6.12	5.52	5.49	5.41	4.91
*p*-Value		0.014*	0.001*	0.002*	0.106	0.027*	0.0002*	<0.0001*	<0.0001*	<0.0001*
STDEV	2.91	3.37	2.95	3.09	3.41	3.32	3.61	3.66	3.42	3.90
MID	1.45									

*(A) Mean IPSS-I patient-reported scores at baseline and during follow-up*.

*(B) Mean IPSS-I patient-reported scores with baseline IPSS score <8 for 102 patients*.

*(C) Mean IPSS-I patient-reported scores with baseline IPSS score ≥8 for 102 patients*.

**Figure 3:**
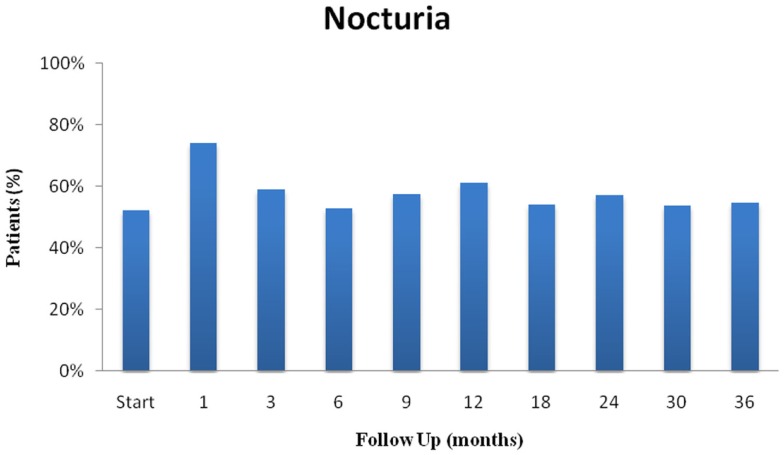
**Nocturia following SBRT for prostate cancer: (nocturia was defined as urinating ≥2 times per night)**.

Individual irritative voiding symptoms (frequency, urgency, and nocturia) followed a similar trend (Table 3). At 1 month post-SBRT, frequency significantly increased (*p* < 0.0001), but returned to baseline at 3 months (*p* = 0.14) (Table 3A). By 2 years, frequency had actually declined to below baseline (*p* = 0.002). Likewise, urgency increased significantly at 1 month and returned to baseline at 3 months (*p* = 0.64) (Table 3B). However, a second late protracted increase in urgency occurred between 9 and 18 months. Urgency returned to near baseline by 2 years post-SBRT. Similarly, nocturia increased transiently at 1 month and then again at 12 months (Table 3C; Figure 3). The pre-treatment and 3-year nocturia rates were similar.

**Table 3:** **Patient-reported responses to IPSS-irritative voiding questions at baseline and following SBRT for prostate cancer, recorded as the percent of the patient cohort**.

	Start	1	3	6	9	12	18	24	30	36
**A. Frequency**
Never	17.7%	11.5%	17.7%	16.1%	13.5%	17.4%	15.8%	21.7%	19.3%	21.7%
Less than half	52.7%	44.5%	62.6%	60.2%	57.8%	51.7%	60.6%	57.7%	58.5%	52.2%
Half or more	24.1%	37.5%	16.7%	21.5%	23.2%	27.0%	21.2%	16.0%	19.3%	23.6%
Almost always	5.4%	6.5%	3.0%	2.2%	5.4%	3.9%	2.4%	4.6%	2.9%	2.5%
Wilcoxon		<0.0001*	0.1358	0.1787	0.8268	0.9429	0.093	0.0022*	0.0066*	0.1198
*N* =	*203*	*200*	*198*	*186*	*185*	*178*	*165*	*175*	*171*	*157*
**B. Urgency**
Never	41.9%	21.0%	35.5%	31.2%	31.4%	32.0%	33.5%	39.4%	35.1%	39.5%
Less than half	41.9%	47.0%	47.2%	50.5%	48.6%	45.5%	48.2%	42.3%	46.8%	43.3%
Half or more	10.8%	25.0%	12.2%	14.5%	13.5%	16.3%	12.2%	11.4%	12.9%	10.2%
Almost always	5.4%	7.0%	5.1%	3.8%	6.5%	6.2%	6.7%	6.9%	5.3%	7.0%
Wilcoxon		<0.0001*	0.6357	0.0943	0.0738	0.0287*	0.1207	0.6797	0.6818	0.5435
*N* =	*203*	*200*	*198*	*186*	*185*	*178*	*165*	*175*	*171*	*157*
**C. Nocturia**
None	9.4%	5.0%	9.6%	7.5%	7.6%	10.1%	9.1%	9.1%	9.9%	6.4%
1 time	38.4%	21.0%	31.3%	39.8%	35.1%	28.7%	37.0%	33.7%	36.3%	38.9%
2 times	25.6%	31.0%	34.8%	29.0%	28.6%	34.3%	30.3%	32.6%	30.4%	33.8%
≥3 times	26.6%	43.0%	24.2%	23.7%	28.6%	27.0%	23.6%	24.6%	23.4%	21.0%
Wilcoxon		< 0.0001**	0.5314	0.5303	0.2733	0.087	0.9624	0.6498	0.797	0.8063
*N* =	*203*	*200*	*198*	*186*	*185*	*178*	*165*	*175*	*171*	*157*

*(A) Frequency (IPSS question 2) defined as how often a patient had to urinate within 2 hours of previously urinating*.

*(B) Urgency (IPSS question 4) defined as how often a patient found it difficult to postpone urination*.

*(C) Nocturia (IPSS question 7) defined as how often a patient needed to get up to urinate at night*.

Next, we assessed the impact of SBRT on irritative voiding symptoms 3 years following treatment on men with baseline mild (IPSS < 8, 102 men) LUTS versus moderate to severe (IPSS ≥ 8, 102 men) LUTS (Tables 2B,C; Figures 1B,C) (32). In men with mild LUTS at baseline, the mean IPSS-I returned to near baseline by 3 years post-SBRT (Table 2B; Figure 1B). In men with moderate to severe LUTS at baseline, the mean IPSS-I significantly decreased from a baseline score of 6.8 to 4.9 at 3 years post-SBRT (Table 2C; Figure 1C). This decrease was both statistically (*p* < 0.0001) and clinically significant (MID = 1.45).

At baseline, 63.1% of our cohort reported some level of bother due to urinary frequency with 17.7% of patients feeling it was a moderate to big problem (Table 4). At 1 month post-SBRT, moderate to big bother with urinary frequency increased to 28% (*p* < 0.0001), but reduced to 15.2% at 3 months (*p* = 0.7251). Although bother declined quickly, a second late transient increase in bother occurred at 12 months (Table 4). Despite this increase, only 14.6% of patients felt that weak urine stream and/or incomplete emptying was a moderate to big problem at 3 years post-SBRT (*p* = 0.2303).

**Table 4:** **Bother with frequency at baseline and following SBRT for prostate cancer (question 4e of the EPIC-26)**.

	**Start**	**1**	**3**	**6**	**9**	**12**	**18**	**24**	**30**	**36**
No problem	36.9%	17.5%	35.9%	34.9%	32.4%	31.5%	39.4%	42.3%	39.8%	42.0%
Very small–small problem	45.3%	54.5%	49.0%	50.0%	50.3%	48.9%	44.8%	44.0%	44.4%	43.3%
Moderate—big problem	17.7%	28.0%	15.2%	15.1%	17.3%	19.7%	15.8%	13.7%	15.8%	14.6%
Wilcoxon		< 0.0001*	0.7251	0.9337	0.4439	0.1663	0.6522	0.1859	0.5768	0.2303
*N* =	*203*	*200*	*198*	*186*	*185*	*178*	*165*	*175*	*171*	*157*

*Patients were stratified into three groups: no problem, very small to small problem, and moderate to big problem. The percentage of patients in each group at each time point is depicted in the chart over 3 years*.

## Discussion

Urinary toxicity following prostate radiotherapy involves both obstructive and irritative symptoms. Irritative voiding symptoms are more bothersome (4), yet they remain understudied. A better understanding of the pattern of irritative voiding symptoms following SBRT would enable clinicians to provide more realistic expectations to patients (33). In this study, we utilized validated QoL questionnaires to comprehensively evaluate irritative voiding symptoms following SBRT (27, 34).

An acute increase in irritative voiding symptoms occurs in most patients post-SBRT. It is believed to occur secondary to inflammation of the bladder neck/urethra. This study shows that SBRT acutely increases all irritative voiding symptoms (frequency, urgency and nocturia) in a similar manner. Furthermore, our results appear comparable to those reported for IMRT and brachytherapy (4). Irritative voiding symptoms may occur secondary to detrusor overactivity and could be treated as such (35). However, antimuscarinics are not routinely prescribed at our institution due to their known side effects and the potential risk of increased post-void residuals (36). Alternative approaches to managing acute irritative voiding symptoms post-SBRT should be explored.

Nocturia is the most bothersome LUTS (37). Nocturnal voiding as little as twice per night is associated with decreased quality of life and may increase the risk of falls (11–13) To prevent falls and their associated morbidity, patients should be educated about their increased incidence following prostate radiation therapy. This study confirms the high incidence of baseline nocturia in elderly men with prostate cancer (38), while also showing a transient increase in nocturia at one month and twelve months post-treatment.

Due to its effectiveness and convenience, brachytherapy is a common treatment option for prostate cancer. Post-implant irritative voiding symptoms are a common toxicity that may impact long-term quality of life. IPSS resolution following brachytherapy varies from months to years (29, 39, 40). Our mean IPSS-I scores returned to baseline within 3 months post-SBRT. A minority of patients experienced a transient increase in irritative voiding symptoms greater than six months after the completion of SBRT. As with brachytherapy, late urinary symptom flare (41–43) occurred in a minority of our patients and resolved with conservative management. Knowledge of this late transient increase in irritative voiding symptoms will enable clinicians to address patient concerns.

Bother is defined as the degree of interference or annoyance caused by a symptom (39, 44). Similar to other radiation modalities, bother with urinary frequency plateaued at one month following treatment with 28% of men reporting it to be a moderate to big problem. This change compares favorably to the change reported at two months with conventionally fractionated EBRT (34%) and brachytherapy (45%) (4). As seen with EBRT, this increase in bother was transient and returned to baseline by 3 months post-SBRT. A second increase in bother occurred twelve months post-SBRT with 19.7% of patients reporting moderate to big bother at this time point. This change is comparable to that reported at twelve months with brachytherapy (20%) (4). Unlike brachytherapy, bother following SBRT returned to near baseline by 2 years (7.1 vs. 20%) and improved over baseline at 3 years.

Compared with prostatectomy, radiation therapy causes less incontinence at the expense of increased acute irritative voiding symptoms. However, this study shows that SBRT, like radical prostatectomy (45), may prevent age dependent increases in late irritative voiding symptoms. As seen following prostatectomy, men with moderate to severe LUTS benefited the most (45). Radical prostatectomy has been shown to improve irritative voiding symptoms within the first year following treatment (4, 46, 47). It has been hypothesized that this is secondary to relief of prostatic obstruction. SBRT is ablative, with a decrease in prostate size seen within the first 3 years following treatment (22). Etiology of reduced irritative voiding symptoms 3 years post-SBRT is unclear, but may be due to prostate size reduction associated with SBRT treatment.

There were several limitations to this study. The EPIC-26 assesses bother associated with frequency but not bother related to urgency and nocturia (30). However, due to the high correlation between bother associated with these symptoms, it is unlikely that this would impact our conclusions. In addition, alternative mechanisms could explain the late improvement in irritative voiding symptoms such as prostate size reduction secondary to ADT and increased alpha antagonist usage (48). However, ADT usage was unlikely the main cause of improvement, as <14% of patients received it with a median duration of only 3 months and a total of three patients, 1.5%, receiving ADT for longer than 6 months. In addition, the baseline QOL assessment took place prior to treatment, on the first day of SBRT, 3 months after the initiation of ADT (49). Likewise, pre-treatment alpha antagonist usage was high and returned to baseline by 2–3 years post-SBRT (50).

## Conclusion

Stereotactic body radiation therapy treatment resulted in an acute increase in irritative voiding symptoms that peaked at one month post-treatment. These symptoms returned to baseline in the majority of patients by 3 months. Patients with moderate to severe LUTS can expect an improvement in their baseline irritative voiding symptoms years after treatment. Bother with urinary frequency was at baseline 2 years post-SBRT and improved by 3 years post-SBRT.

## Conflict of Interest Statement

Sean P. Collins and Brian T. Collins serve as clinical consultants to Accuray Inc. The Department of Radiation Medicine at Georgetown University Hospital receives a grant from Accuray to support a research coordinator. The other authors declare that they have no competing interests.

## Abbreviations

ADT, androgen deprivation therapy; BED, biologically effective dose; BPH, benign prostatic hyperplasia; CT, computed tomography; CTV, clinical target volume; DVH, dose-volume histogram; EPIC, expanded prostate index composite; EQD2, equivalent dose in 2-Gy fractions; GTV, gross target volume; Gy, gray; IMRT, intensity modulated radiation therapy; IPSS, international prostate symptom score; IRB, institutional review board; LUTS, lower urinary tract symptoms; MID, minimally important difference; MR, magnetic resonance; PTV, planning target volume; QoL, quality of life; SD, standard deviation; SBRT, stereotactic body radiation therapy.
